# Zinc in Wheat Grain, Processing, and Food

**DOI:** 10.3389/fnut.2020.00124

**Published:** 2020-08-18

**Authors:** Min Wang, Fanmei Kong, Rui Liu, Qingqi Fan, Xiaocun Zhang

**Affiliations:** ^1^College of Food Science and Engineering, Shandong Agricultural University, Tai'an, China; ^2^College of Resources and Environment, Shandong Agricultural University, Tai'an, China; ^3^Institute of Food and Nutrition Development, Ministry of Agriculture and Rural Affairs, Beijing, China; ^4^Crop Research Institute, Shandong Academy of Agricultural Sciences, Jinan, China

**Keywords:** wheat, zinc, flour, fortification, bioavailability

## Abstract

Improving zinc (Zn) content in wheat and its processed foods is an effective way to solve human Zn deficiency, which can cause a variety of diseases. This article summarizes the works on Zn in wheat grain, wheat processing, and wheat-derived foods. Grain Zn content in wheat was 31.84 mg·kg^−1^ globally but varied across continents, for example, 25.10 mg·kg^−1^ in Europe, 29.00 mg·kg^−1^ in Africa, 33.63 mg·kg^−1^ in Asia, and 33.91 mg·kg^−1^ in North America. Grain Zn content in wheat improved from 28.96 to 36.61 mg·kg^−1^ and that in flour increased from 10.51 to 14.82 mg·kg^−1^ after Zn fortification. Furthermore, Zn content varied in the different processed components of wheat; that is, Zn content was 12.58 mg·kg^−1^ in flour, 70.49 mg·kg^−1^ in shorts, and 86.45 mg·kg^−1^ in bran. Zinc content was also different in wheat-derived foods, such as 13.65 mg·kg^−1^ in baked food, 10.65 mg·kg^−1^ in fried food, and 8.03 mg·kg^−1^ in cooking food. Therefore, the suitable Zn fortification, appropriate processing, and food type of wheat are important to meet people's Zn requirement through wheat.

## Introduction

Zinc is a blue-white metal element that accounts for ~0.02% of the earth's crust and is the twenty-third largest element in abundance (1). Zinc is an essential trace element for the growth and development of humans (2), animals (3), and plants (4). The health benefits of Zn have received much attention since the 1960s. According to the Zn intake reference of the World Health Organization (WHO), the recommended daily Zn intake of infants aged 7–12 months is 5 mg·day^−1^, children aged 1–10 years is 10 mg·day^−1^, males and females aged 11–51 years is 15 mg·day^−1^, and pregnant women is 20–25 mg·day^−1^, and the daily Zn tolerance is 100 mg·day^−1^. However, at least 25% of the global population is at risk of Zn deficiency (5). Insufficient Zn intake can cause loss of appetite, growth retardation, rough and peeling skin, and immune system dysfunction (6). On the one hand, Zn deficiency in pregnant women may cause fetal malformations (7), and nearly 82% of pregnant women worldwide have insufficient Zn intake (5). On the other hand, excessive Zn intake can cause nausea, vomiting, lethargy, and fatigue. Therefore, maintaining the balance of Zn in the human body, including increasing Zn intake for people with Zn deficiency, is an important long-term task.

Wheat is one of the major grains worldwide, which provides nearly 20% calorie and protein per capita worldwide (8). Therefore, improving the daily Zn intake through wheat-derived processed foods is an important way to solve Zn deficiency.

Grain Zn content in wheat is usually low in several areas and is therefore the first and essential concern in this study. Wheat processing is another important factor that can remarkably affect the actual Zn intake of people because the aleurone and bran layers of wheat are usually removed during processing. Different processing technologies of wheat also lead to a large loss of Zn (9). In addition, the content of phytic acid and the molar ratio of phytic acid to Zn, which can affect Zn absorption, are among the important factors to be considered.

This article summarizes the results in the list of fields in recent years, including (1) zinc content in wheat, wheat processing, and wheat-derived foods and (2) the bioavailability of Zn in wheat.

## Grain Zinc Content in Wheat

Wheat is one of the important sources of daily diet in developing countries, but its Zn content is relatively low. The Zn content of wheat grains need to reach 45.00 mg·kg^−1^ to meet the Zn needs of the human body (10). However, statistics show that the average Zn content in wheat grains worldwide is only 28.48 mg·kg^−1^, which is lower than the internationally recommended amount. The grain Zn content of wheat in different countries since the 1960s (Table S1) ranged from 8.00 to 88.20 mg·kg^−1^ with an average value of 31.84 mg·kg^−1^ (5, 9–47).

**Figure 1:**
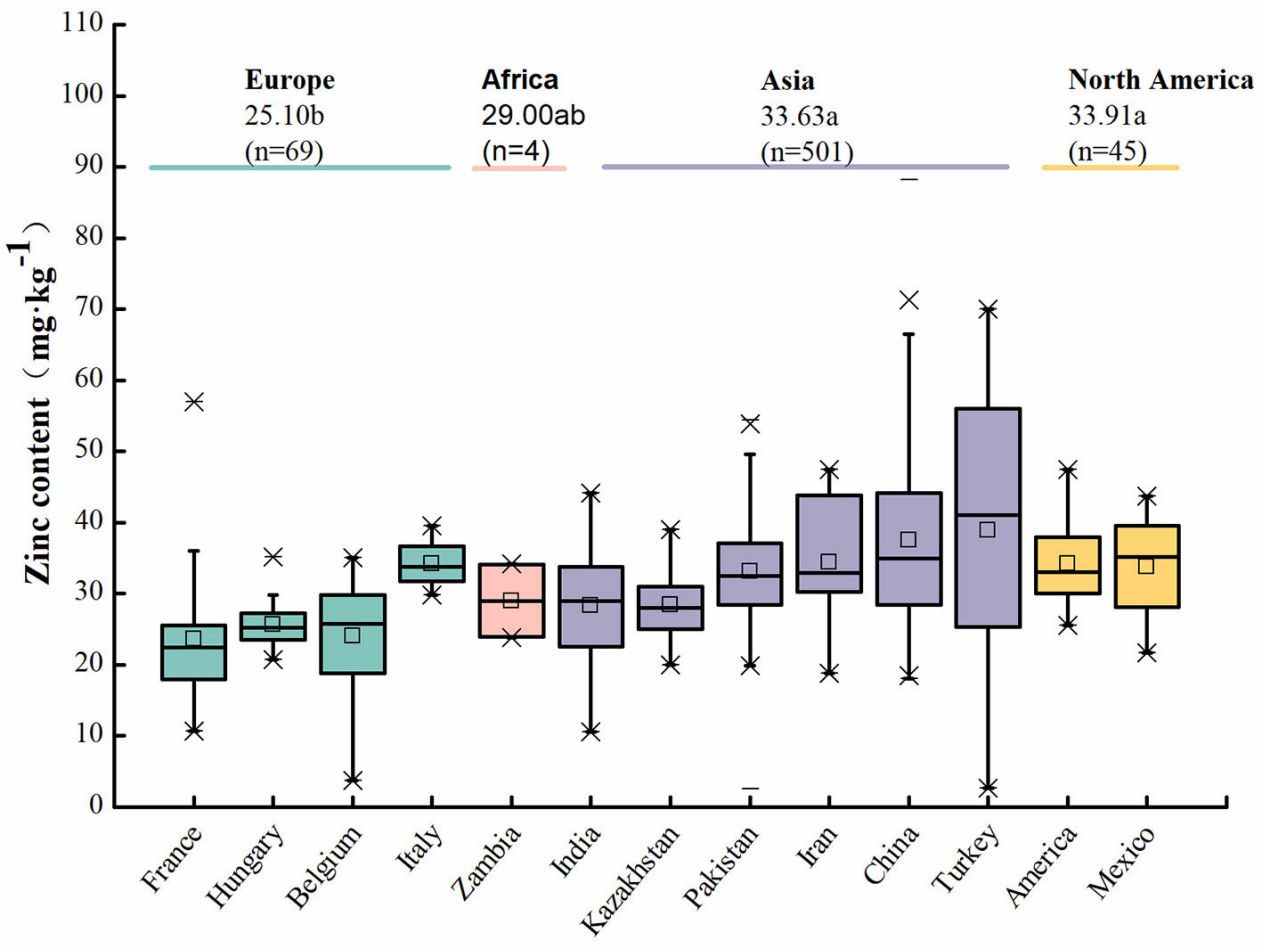
Zinc content of wheat among different countries and continents. These data come from references (5, 9–47). Letters a and b indicate a significant difference at the level of *P* < 0.05.

The grain Zn contents of wheat among different countries varied (Figure 1). For example, grain Zn content is 38.87 mg·kg^−1^ in Turkey, 37.51 mg·kg^−1^ in China, 34.43 mg·kg^−1^ in Iran, 34.21 mg·kg^−1^ in the United States, 34.20 mg·kg^−1^ in Italy, 33.76 mg·kg^−1^ in Mexico, 33.13 mg·kg^−1^ in Pakistan, 29.00 mg·kg^−1^ in Zambia, 28.40 mg·kg^−1^ in Kazakhstan, 28.31 mg·kg^−1^ in India, 25.68 mg·kg^−1^ in Hungary, 24.09 mg·kg^−1^ in Belgium, and 23.58 mg·kg^−1^ in France. The average grain Zn contents of wheat among different continents were 25.10 mg·kg^−1^ in Europe, 29.00 mg·kg^−1^ in Africa, 33.63 mg·kg^−1^ in Asia, and 33.91 mg·kg^−1^ in North America. The reasons for these differences in grain Zn content may be as follows: (1) The amount of Zn available in the soil in these areas is different. For example, the available Zn in the soil in China was 0.37 mg·kg^−1^, 0.53 mg·kg^−1^ in India, 0.55 mg·kg^−1^ in Pakistan, and 1.46 mg·kg^−1^ in Zambia (12). (2) Grain Zn content is different among wheat varieties. For example, the grain Zn content was 28.48 mg·kg^−1^ in common wheat, 34.80 mg·kg^−1^ in white wheat, 34.40 mg·kg^−1^ in red wheat, and 36.45 mg·kg^−1^ in black wheat (13). (3) Different wheat cultivation types, environments, climates, and biofortification methods result in the different grain Zn contents of wheat. For example, the mean Zn content was 31.42 mg·kg^−1^ in winter wheat and 30.13 mg·kg^−1^ in spring wheat (Figure S1).

Zinc content can be enhanced by fortification during breeding and by adding nutrients during food processing (48). Biofortification can quickly increase grain Zn content in wheat because of agronomic measures, such as the application of Zn fertilizer (49); therefore, field fertilization was gradually accepted and liked by farmers. The main application methods of Zn fertilizer include soil application, foliar application, and seed treatment (50). As shown in Figure S1, grain Zn content in wheat by soil Zn application ranged from 8.00 to 54.40 mg·kg^−1^, with an average value of 29.39 mg·kg^−1^ (5, 9, 10, 12, 14, 19, 22, 25, 27, 28, 30, 31, 34, 35, 51); that by foliar fertilization ranged from 25.1 to 88.20 mg·kg^−1^, with an average value of 42.30 mg·kg^−1^ (5, 15, 18, 20, 22, 31, 32); and that by seed soaking treatment ranged from 25.70 to 31.10 mg·kg^−1^, with an average value of 30.30 mg·kg^−1^ (19, 41). Furthermore, grain Zn content by the combined treatment of soil application and foliar fertilization ranged from 25.10 to 70.00 mg·kg^−1^ with an average value of 40.45 mg·kg^−1^ (5, 22, 25, 31, 33), and that by the combined treatment of soil fertilization and seed soaking reached 34.30 mg·kg^−1^ (19).

**Figure 2:**
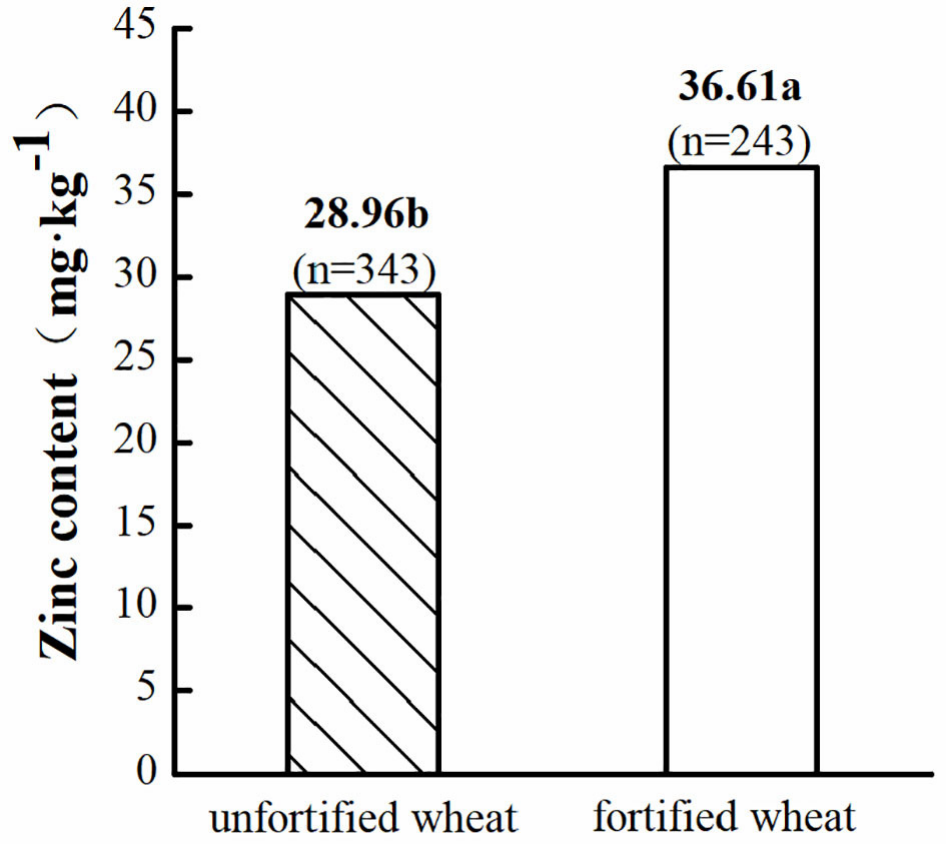
Zinc content in fortified and unfortified wheat. Letters a and b indicate a significant difference at the level of *P* < 0.05; *n* is the total number of samples.

In general, the average grain Zn content in wheat without Zn fertilizer was 28.96 mg·kg^−1^, which increased to 36.61 mg·kg^−1^ after Zn fortification (Figure 2). Interestingly, the available Zn concentration in soil could exceed 4.09 mg·kg^−1^, and grain Zn content could reach 40–60 mg·kg^−1^ (52) when 50 kg·hm^−2^ ZnSO_4_·7(H_2_O) was added to the soil.

Therefore, the most effective way to increase grain Zn content in wheat may be combining soil fertilization and foliar fertilization or directly applying foliar fertilization. However, local environment and wheat varieties should also be considered when choosing a suitable fertilization method to increase grain Zn content in wheat.

## Zinc in Different Components of Wheat

The grain structure of wheat is generally divided into bran, embryo, and endosperm, which account for 14–16, 2–3, and 81–84% of the grain, respectively (53). Wheat peeling is the process of separating the endosperm and bran of wheat and grinding them into flour (28). Wheat flour processing has two purposes: one is to grind the endosperm into small and fine particles, and the other is to remove the bran as much as possible. The traditional method of flour making breaks wheat directly to separate the bran and endosperm and obtains the pure wheat core through flour cleaning, which is further ground into flour. The other milling method carries out the peeling process before wheat milling. Generally, wheat peeling is carried out by a friction machine or scraping machine equipment. A friction machine mainly carries out peeling through friction between wheat grains. The scraping machine uses a sand roller to scrape off wheat bran (54). According to reported results since 1980s (Table S2), the basic law of Zn content in wheat components after milling is: bran > shorts > flour. Zinc content over the years and across countries ranged from 3.73 to 36.53 mg·kg^−1^ in flour with an average value of 12.58 mg·kg^−1^ (22, 23, 29, 37, 38, 42, 45, 55–69), ranged from 26.60 to 139.84 mg·kg^−1^ in shorts with an average of 70.49 mg·kg^−1^ (29, 38, 42, 56, 59, 62, 66, 70), and ranged from 23.80 to 159.80 mg·kg^−1^ in bran with an average of 86.45 mg·kg^−1^ (21, 22, 29, 32, 38, 42, 46, 56, 59, 61, 62, 66, 68, 70).

The results showed that Zn was mainly concentrated in the cortex and embryo of wheat grain. For example, Zn content in bran was about three times higher than that in endosperm (71, 72) and approximately six times higher than that in flour after milling (42, 70).

**Figure 3:**
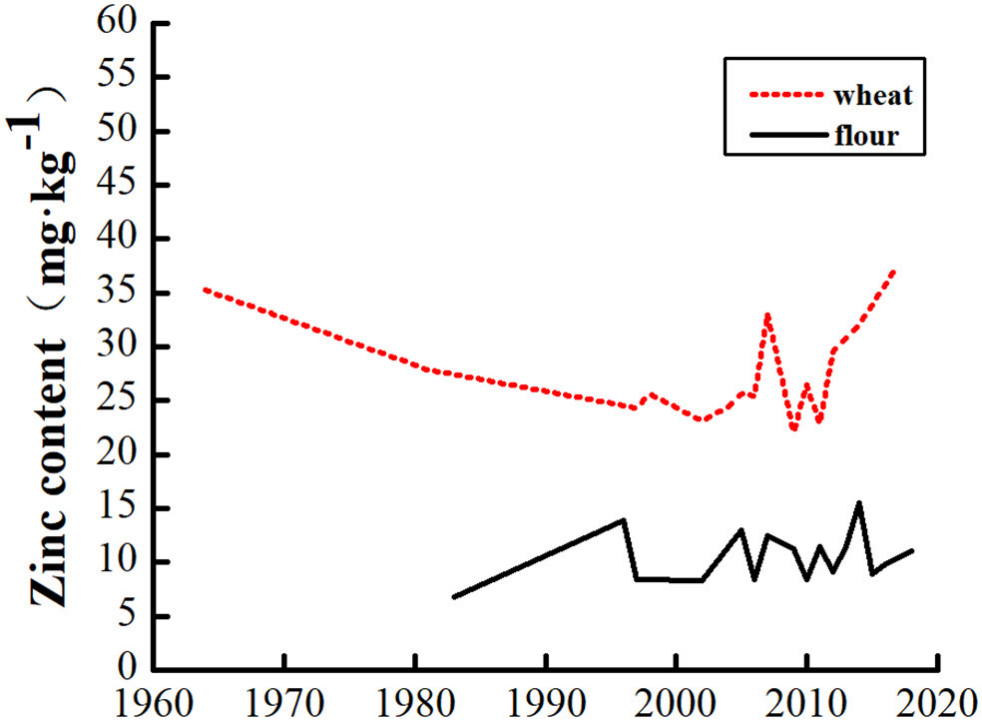
Zinc content in wheat grain and flour with year.

**Figure 4:**
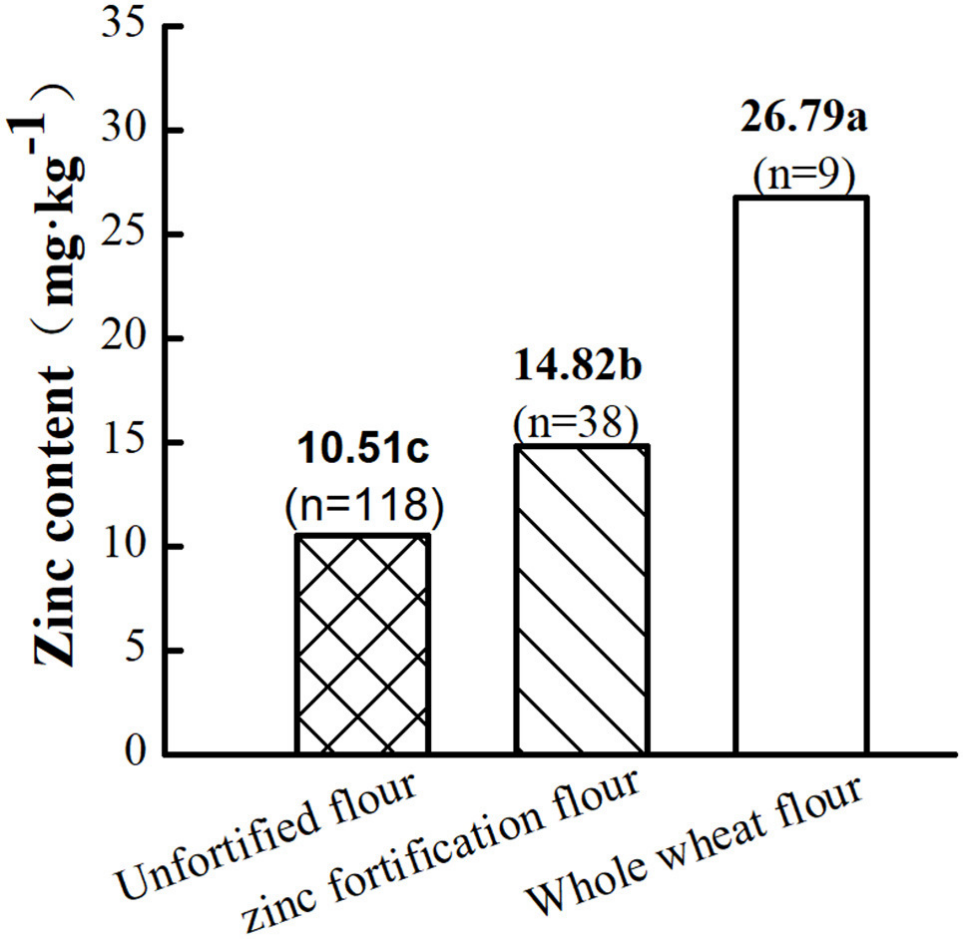
Comparison of wheat flour with different treatments. Data are from references (22, 23, 29, 37, 38, 42, 45, 55–69). Letters a, b, and c indicate a significant difference at the level of *P* < 0.05; *n* is the total number of samples.

The overall trend of grain and flour Zn content gradually increased with year (Figure 3). In particular, grain and flour Zn content increased remarkably since the launch of the Harvest Plus project in 2003 possibly owing to biological strengthening, such as Zn fertilization. Flour Zn content could reach 14.82 mg·kg^−1^ after Zn fortification but was only 10.51 mg·kg^−1^ in unfortified flour and 26.79 mg·kg^−1^ in whole wheat flour (Figure 4).

**Figure 5:**
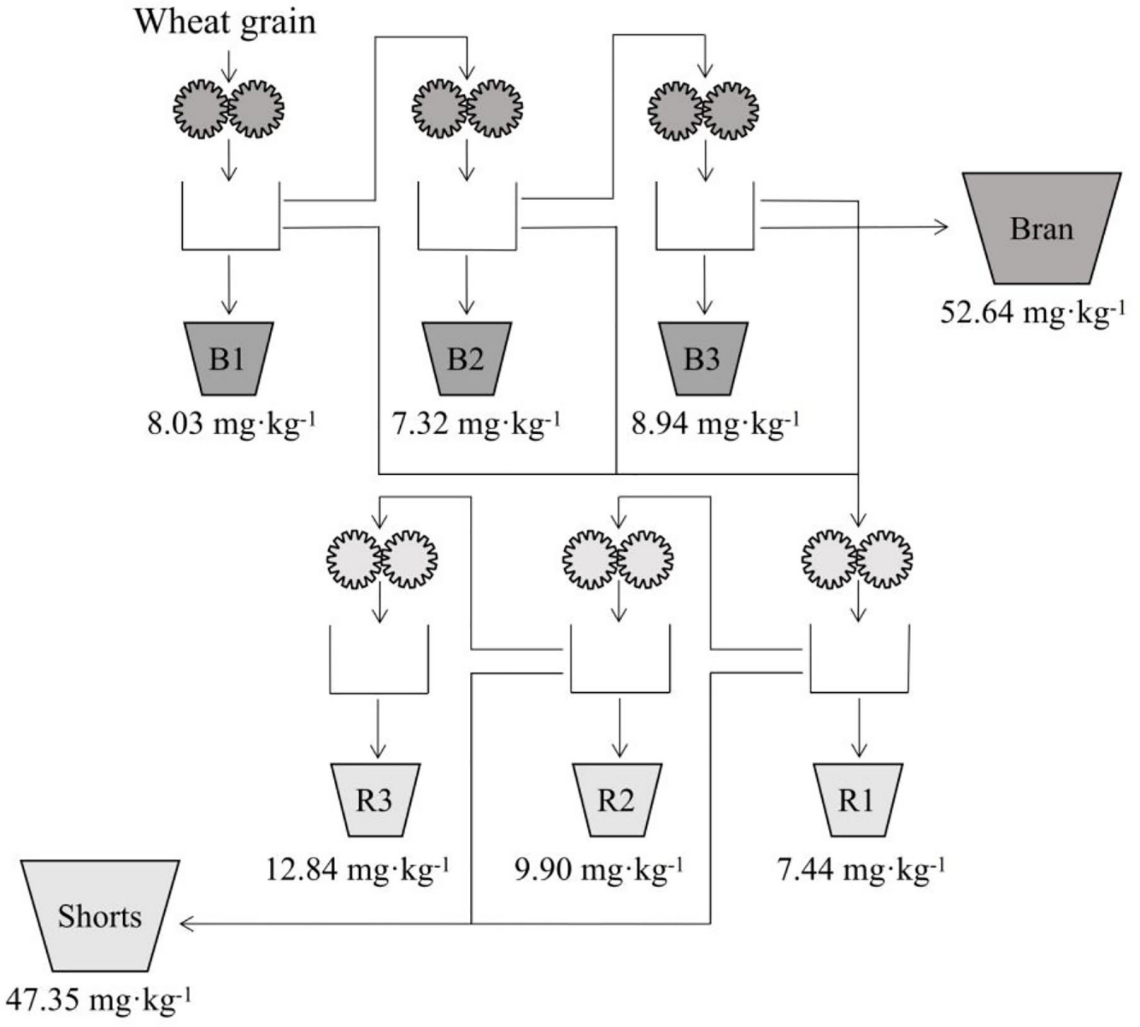
Schematic diagram of zinc content in the powder path. These data are from references (38, 59, 70).

The technological process of wheat milling is called “flour road.” Eight milling parts can be obtained after wheat milling, including six flour parts [i.e., three “broken” (B) and three “reduced” (R) milling parts] and two kinds of bran parts (i.e., bran and shorts). The flour collected by B1, R1, B2, R2, B3, and R3 is standard flour; that by B1, R1, B2, and B3 is bread flour; and that by B1 and R1 is refined flour (59). The powder path schematic diagram of Bühler MLU 202 mill and the Zn content of each powder path after passing the wheat grain through the mill are shown in Figure 5. The Zn content in each component was in the following order: bran > R (powder) > B (powder). Zinc content was 12.84 mg·kg^−1^ in R1, 9.90 mg·kg^−1^ in R2, 7.44 mg·kg^−1^ in R3, 8.03 mg·kg^−1^ in B1, 8.94 mg·kg^−1^ in B2, and 7.32 mg·kg^−1^ in B3. The Zn content in bran was remarkably higher than that of the other components. So the processing with higher flour extraction rate including aleurone layer would enhance retention of more Zn. However, this may also affect overall flour quality (57).

In summary, Zn content in flour can be increased in two ways. One is to increase grain Zn content through agronomic treatments. However, the enhancement of the target on a large scale by field fertilization is difficult to achieve (Table S1), and long-term fertilization may also cause pollution to the soil and other environments. The other way is to choose an appropriate wheat processing. This approach may be a direct way to reach Zn reference target by whole wheat flour. Zinc was not lost in flour; however, whole wheat flour is rough, its dough is poorly fermented, and the taste of its derived food taste is not good. Safety risks may even occur owing to pesticides and heavy metals in the seed coat (73). Thus, Zn-rich flour from aleurone flour mixed with ordinary flour in a certain proportion was used to meet the standards of first-grade flour, and this flour had no effect on food's taste despite the addition of 5% aleurone flour (74, 75). We also could decrease the influence of pesticides and heavy metals while maintaining Zn content by controlling the peeling rate and flour extraction rate. For example, the Zn content of flour could reach 19.40 mg·kg^−1^, which exceeds the fortification standard of 10.00 mg·kg^−1^ when flour extraction rate was 65–75% (61).

Therefore, we should pay attention to improve grain Zn content and also consider wheat processing to control the peeling rate while keeping Zn content to a suitable level.

## Zinc in Wheat Processed Foods

Foods from processed wheat, such as bread, steamed buns, and biscuits, are the main traditional staple food that occupy an important position in people's dietary structure (76). For example, wheat-related traditional foods in China account for 75% of China's total wheat consumption (77).

Table 1 shows a list of the Zn content of processed foods from wheat in the past 15 years. The Zn content of processed wheat foods ranged from 1.73 to 53.05 mg·kg^−1^ with an average of 12.56 mg·kg^−1^ (16, 37, 55, 58, 60, 67, 78–86). The processed wheat food with the highest Zn content was steamed buns, which has a Zn content of 53.05 mg·kg^−1^. Cooked noodles have the lowest Zn content, which was only 1.73 mg·kg^−1^.

**Table 1:** Zinc content in different wheat processed foods.

**Name**	**Zinc content (mg·kg**^****−1****^**)**		**References**	**Remark**
	**Mean**	**Range**		
Bread	7.30	NS	Ma et al. (67)	NS
	12.50	NS	Ma et al. (67)	Whole wheat
	16.00	11.70–18.40	Umeta et al. (78)	NS
	4.80	NS	Shi et al. (79)	NS
	7.20	NS	Shi et al. (79)	Zinc-rich yeast
	7.57	7.24–7.81	Ren et al. (80)	NS
	15.04	14.50–15.62	Ren et al. (80)	Adding organic tea
	17.50	14.00–22.00	Bai et al. (81)	NS
	10.00	8.90–11.60	Lazarte et al. (55)	White bread
	18.10	9.30–23.30	Heshe et al. (60)	Adding bran
	26.70	25.60–28.50	Saha et al. (16)	NS
	35.20	NS	Ciccolini et al. (58)	Whole wheat
	6.07	NS	Ciccolini et al. (58)	NS
	10.60	6.40–17.50	Shokunbi et al. (82)	White bread
	13.40	10.80–16.00	Shokunbi et al. (82)	Whole wheat
	13.60	13.40–13.70	Shokunbi et al. (82)	Malt
Steamed buns	6.00	NS	Ma et al. (67)	NS
	5.30	NS	Ma et al. (67)	NS
	8.50	NS	Ma et al. (67)	NS
	28.26	16.05–37.91	Wang (83)	Adding bran
	45.73	25.75–53.05	Wang (83)	Adding bran + zinc fertilizer
Noodles	5.70	NS	Ma et al. (67)	Fresh noodles
	17.70	11.80–22.80	Lazarte et al. (55)	Fresh noodles
	3.69	1.37–6.32	Shokunbi et al. (82)	Dry noodles
	10.10	3.61–21.6	Shokunbi et al. (82)	Adding eggs
Biscuits	11.82	5.57–18.36	Sebecic et al. (84)	NS
	7.10	NS	Ma et al. (67)	NS
	8.30	5.00–11.00	Bai et al. (81)	Adding soda
	12.40	5.00–40.00	Bai et al. (81)	Crisp
Spaghetti	7.50	NS	Ma et al. (67)	NS
	12.90	10.3–15.4	Cubadda et al. (37)	Fresh spaghetti
	11.70	10.70-13.70	Cubadda et al. (37)	Fresh spaghetti
	4.70	4.40–5.20	Cubadda et al. (37)	Dry spaghetti
	4.56	4.31–4.81	Shokunbi et al. (82)	Dry spaghetti
	5.38	5.14–5.61	Shokunbi et al. (82)	Dry spaghetti
Instant noodles	5.50	NS	Ma et al. (67)	NS
	15.70	NS	Zhu et al. (85)	NS
	10.60	NS	Chen et al. (86)	NS
Pancakes	4.70	NS	Ma et al. (67)	NS
	5.80	NS	Ma et al. (67)	NS
Wheat Gluten	27.50	NS	Ma et al. (67)	NS
	19.80	NS	Ma et al. (67)	NS
Cake	11.70	9.00–20.00	Bai et al. (81)	NS
Mean	12.56	1.37–53.05	–	–

*NS indicates that relevant information is not mentioned in the literature*.

**Figure 6:**
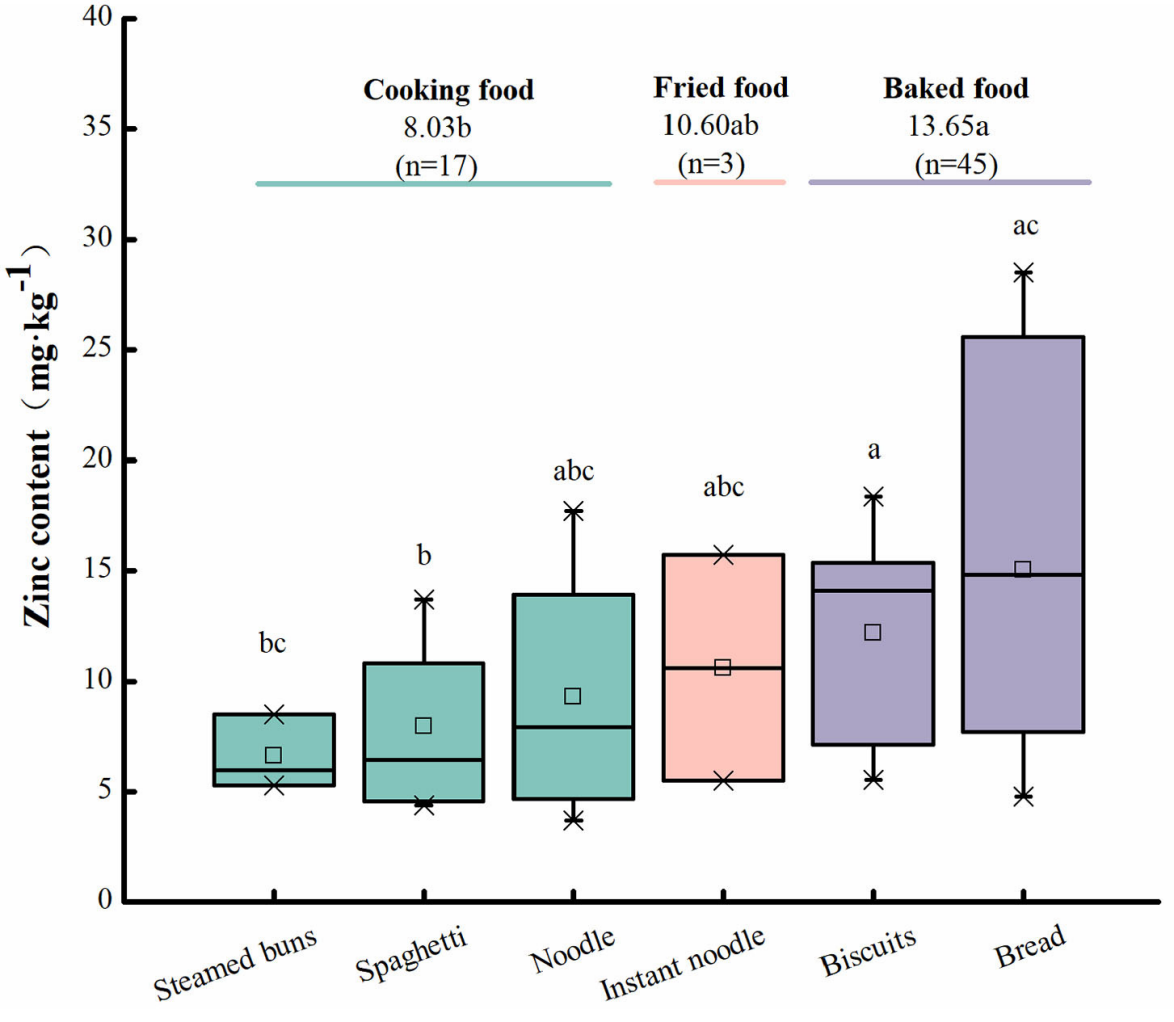
Zinc content in different wheat processed foods. Letters a, b, and c indicate a significant difference at the level of *P* < 0.05; *n* is the total number of samples.

Zinc content was remarkably different among processed wheat foods. For example, Zn content was 15.03 mg·kg^−1^ in bread, 12.20 mg·kg^−1^ in biscuits, 10.60 mg·kg^−1^ in instant noodles, 9.30 mg·kg^−1^ in noodles, 7.94 mg·kg^−1^ in spaghetti, and 6.60 mg·kg^−1^ in steamed buns (Figure 6). The Zn contents of different foods are in the following order: baked food (13.65 mg·kg^−1^) > fried food (10.60 mg·kg^−1^) > cooking food (8.03 mg·kg^−1^). Baked foods have a remarkably higher Zn content than cooking foods and fried foods. This difference in Zn content may be ascribed to the following: (1) The Zn content of the flour used in these foods was different and ranged from 3.73 to 36.53 mg·kg^−1^. For example, the Zn content of unfortified flour was 10.51 mg·kg^−1^, whereas that of Zn-fortified flour and whole wheat flour was 14.82 and 26.79 mg·kg^−1^, respectively (Figure 4). Meanwhile, these foods require different kinds of flour, in which protein contents often vary. For example, in the same growth environment, protein content of strong gluten wheat could reach 148.86 mg·kg^−1^, weak gluten wheat was only 108.51 mg·kg^−1^, and middle gluten wheat was 137.41 mg·kg^−1^ (87). Zn mainly binds to proteins in plants. So protein content may affect the amount of Zn binding, which indirectly affects Zn content. (2) Different food processing methods might also affect zinc content. For example, fried foods usually need oil, and cooking food need water. Yet Zn dissolves more easily in water than in oil, which might result in more Zn loss in cooking food processing than fried foods processing (88).

## Zinc Bioavailability in Wheat

Bioavailability refers to the ratio of an animal's ingested nutrients that can be absorbed by the small intestine and participate in metabolic processes or stored in animal tissues. The bioavailability of Zn in wheat is affected by many factors, of which phytic acid is the main factor. Phytic acid has a strong chelating ability and can be combined with Zn^2+^ in food to form insoluble complexes. This insoluble Zn is difficult to hydrolyze by the human digestive process; therefore, the combination of Zn and phytic acid inhibits Zn absorption. Furthermore, humans lack phytase. Zinc can be linked to phytate; thus, the utilization of Zn in the intestine is reduced (89).

The bioavailability of Zn in wheat is usually expressed as the molar ratio of phytic acid to Zn. Zinc bioavailability is only 10–15% when the molar ratio is >15. Zinc bioavailability is medium and ranges from 30 to 35% when the molar ratio is 5–15. Zinc bioavailability is high at up to 45–55% when the molar ratio is < 5 (90). For instance, if the Zn content in flour is 15.2 mg·kg^−1^ and the phytic acid content is 272 mg·100 g^−1^, then the phytic acid/Zn ratio is 17.7, which means that the Zn bioavailability is low (55).

Wheat processing affects the phytic acid/Zn molar ratio. Peeling can remarkably influence the phytic acid content in flour because phytic acid is mainly distributed in the aleurone layer. The phytic acid content of flour initially increased and then decreased with the increase in peeling rate (63). Researchers also found that the phytic acid content of flour increased remarkably with the increase in flour yield. The increase in phytic acid content in flour was because more wheat cortex and aleurone layer were mixed in flour with the increase in flour yield (20). For example, phytic acid was 158 mg·100 g^−1^ when peeling rate was 4%. However, phytic acid/Zn molar ratio reached the highest value (20.69) when the peeling rate was 10% and had the lowest value (13.86) when the peeling rate was 0 (63).

Different foods also have different levels of Zn bioavailability. For example, white bread has a Zn content of 10.00 mg·kg^−1^, a phytic acid content of 99 mg·100 g^−1^, and phytic acid/Zn molar ratio of 9.8. In contrast, fresh noodles have Zn content and phytic acid content of 17.7 mg·kg^−1^ and 468 mg·100 g^−1^, respectively; and their phytic acid/Zn molar ratio is 27.4 (55). The average content of Zn and phytic acid in 14 kinds of common Chinese processed wheat foods was 9.34 mg·kg^−1^ and 114.20 mg·100 g^−1^, and their average phytic acid/Zn ratio was 14.4. Among these foods, the phytic acid/Zn molar ratios of wheat bread, instant noodles, whole wheat biscuits, and pasta were more than 15 (67).

Adding phytase to food in the market has not been reported until now. However, researchers found that phytase can rapidly degrade phytic acid in flour after adding phytase to flour; thus, phytase increases the bioavailability of Zn (91). Moreover, Zn deficiency can be solved by cultivating wheat with low phytic acid content. However, mutants with low phytic acid may have a reduced grain yield and may even affect human safety (92).

The current methods to improve Zn bioavailability in wheat and its derived foods mainly included two aspects. One was to increase Zn content in grain, flour, and its derived foods. The other was to reduce the inhibitors that reduce Zn bioavailability in processed wheat foods.

## Conclusions and Future Direction

Grain Zn content in wheat worldwide is 31.84 mg·kg^−1^, which was lower than that suggested by the reference of WHO. Zinc fortification is the primary way to increase the grain Zn content of wheat and could be achieved by Zn fertilizer. In general, the average grain Zn content in wheat could reach 36.61 from 28.96 mg·kg^−1^ after applying Zn fertilizer (Figure 2). Grain Zn content could reach 40–60 mg·kg^−1^ and achieve the reference of WHO under 50 kg·hm^−2^ ZnSO_4_·7(H_2_O) treatment.

Wheat milling has remarkable effects on Zn content. For example, Zn contents in flour, shorts, and bran were 12.58, 70.49, and 86.45 mg·kg^−1^, respectively. The change rule of Zn content in different components of wheat is bran > shorts > flour; the change rule of Zn content in each powder path is bran > B flour > M flour, B_3_ > B_1_ > B_2_, and M_3_ > M_2_ > M_1_. However, only a few studies have been conducted in this area. Moreover, Zn content in different wheat-derived foods is remarkably different, for example, 13.65 mg·kg^−1^ in baked food, 10.65 mg·kg^−1^ in fried food, and 8.03 mg·kg^−1^ in cooking food. This difference in Zn content may be due to the method of food processing.

Zinc deficiency is a comprehensive problem. Rotation and other cultivation methods may be used to increase Zn content to a certain extent. For example, the rotation of corn and wheat results in a higher Zn content in the second quarter compared with that in the first quarter (5). Although the application of Zn fertilizer can improve the Zn content, we should also consider the possible pollution of Zn to the environment by long-term application of Zn fertilizer under the premise of achieving the recommended Zn level (19).

Developing wheat varieties with grains that are rich in Zn is a new approach. Conventional wheat breeding and modern biotechnology methods (93), such as transferring a high Zn accumulation gene from wheat relatives, may be promising.

In the future, we should focus on how to preserve Zn as much as possible during milling, such as the effects of wheat grain pre-treatment and milling procedures on flour Zn content. Moreover, we should also explore the impact of food processing methods on Zn content.

Phytic acid is an important indicator that affects the effectiveness of Zn. Current research has focused on phytic acid content in wheat grains and flour. However, research on phytic acid content in wheat-derived foods is scarce. Next, we should strengthen the study of the effects of different processing methods on phytic acid content and Zn bioavailability.

Zinc deficiency is a worldwide problem. Zinc fortification should strengthen international cooperation through international schemes, such as Harvest Plus. Moreover, we should raise public awareness of the dangers of Zn deficiency and obtain as much support as possible, such as sustained funding, from the local government.

Therefore, suitable Zn fortification, appropriate processing, reasonable food type, international collaboration, and government support are important to meet people's Zn requirements through wheat.

## Author Contributions

MW collected and analyzed the data. MW and XZ wrote this paper. XZ, FK, RL, and QF conceived and modified this paper. All authors contributed to the article and approved the submitted version.

## Conflict of Interest

The authors declare that the research was conducted in the absence of any commercial or financial relationships that could be construed as a potential conflict of interest.
