# Supplementary material for: Zinc in Wheat Grain, Processing, and Food
Source: Front Nutr. 2020 Aug 18;7:124. doi: 10.3389/fnut.2020.00124 (PMC7471629; doi:10.3389/fnut.2020.00124)
Supplement: Supplementary file 1 [file Data_Sheet_2.docx]

Supplementary Figures notes

**Figure 1** Zinc content of wheat among different countries and continents

Note: These data comes from the references (5, 10, 13-50); The letters a and b indicate a significant difference at the level of *P* < 0.05.

**Figure 2** Zinc content in fortified and unfortified wheat

Note: The letters a and b indicate a significant difference at the level of P < 0.05; n is the total number of samples.

**Figure 3** Zinc content in wheat grain and flour with year

**Figure 4** Comparison of wheat flour with different treatments

Note: Data from these references (11, 25, 26, 32, 40, 41, 45, 48, 57-71); The letters a and b indicate a significant difference at the level of P < 0.05; n is the total number of samples.

**Figure 5** Schematic diagram of zinc content in the powder path

Note: These data are from the references (41, 60, 71).

**Figure 6** Zinc content in different wheat processed foods

Note: The letters a and b indicate a significant difference at the level of P < 0.05; n is the total number of samples.
