# Supplementary material for: Zinc in Wheat Grain, Processing, and Food
Source: Front Nutr. 2020 Aug 18;7:124. doi: 10.3389/fnut.2020.00124 (PMC7471629; doi:10.3389/fnut.2020.00124)
Supplement: Supplementary file 2 [file Data_Sheet_1.docx]

Supplementary Tables

**Table S1** Zinc content in wheat grains around the world

| **Serial number** | **Country** | **Fertilizing method** | **Fertilizing amount** | **Number of samples** | **Grain Zinc content (mg·kg^-1^)** | | **References** |
| --- | --- | --- | --- | --- | --- | --- | --- |
|  |  |  |  |  | **mean** | **range** |  |
| 1 | United States | NS | NS | 10 | 35.00 | 30.00 ~ 46.00 | *Czerniejewski, 1964*(47) |
| 2 | United States | NS | NS | 23 | 27.90 | 26.10 ~ 31.80 | *Pomeranz, 1983*(69) |
| 3 | China | NS | NS | 10 | 25.50 | 10.70 ~ 57.00 | *Wang, 1998*(45) |
| 4 | France | NS | NS | 2 | 18.30 | 18.10 ~ 18.50 | *Wang, 1998*(45) |
| 5 | Mexico | NS | NS | NS | 35.00 | 25.00 ~ 65.00 | *Monasterio,2000*(44) |
| 6 | Mexico | NS | NS | NS | 26.00 | 22.2 ~ 029.00 | *Daniel, et al., 2003*(43) |
| 7 | Belgium | NS | NS | 14 | 26.44 | 16.70 ~ 35.10 | *Ruibal-Mendieta, et al., 2005*(42) |
| 8 | France | Soil fertilization | NS | 51 | 28.50 | 14.00 ~ 43.00 | *Oury, et al., 2006*(9) |
| 9 | Pakistan | NS | NS | 2 | 23.85 | 19.9 ~ 27.8 | *Harris, 2006*(41) |
|  |  | Seed soaking | 0.05, 0.3% ZnSO_4_·7H_2_O | 2 | 28.40 | 25.70 ~ 31.10 | *Harris, 2006*(41) |
| 10 | Kazakhstan | NS | NS | 66 | 28.00 | 20.00 ~ 39.00 | *Morgounov, et al., 2006*(39) |
| 11 | China | NS | NS | 240 | 29.30 | 19.90 ~ 43.30 | *Zhang, et al., 2007*(40) |
| 12 | China | NS | NS | 43 | 28.60 | 21.20 ~ 34.80 | *Tang, et al., 2008*(38) |
| 13 | Hungary | NS | NS | 26 | 25.70 | 20.70 ~ 35.20 | *Zhao, et al., 2009*(35) |
| 14 | Italy | NS | NS | 3 | 34.30 | 29.80 ~ 39.50 | *Cubadda, et al., 2009*(37) |
| 15 | Iran | NS | NS | 137 | 31.60 | 11.70 ~ 64.00 | *Karami, et al., 2009*(36) |
| 16 | Italy | NS | NS | 84 | 33.90 | 28.5 ~ 46.30 | *Ficco, 2009*(34) |
| 17 | China | Foliar fertilization | NS | 3 | 31.78 | 25.40 ~ 43.10 | *Zhang, et al., 2010*(32) |
|  |  | Foliar fertilization | 1.84 kg·hm^-2^ ZnSO_4_·7H_2_O | 3 | 45.70 | 42.60 ~ 47.40 | *Zhang, et al., 2010*(32) |
| 18 | India | Soil fertilization | NS | NS | 33.60 | 32.60 ~ 34.80 | *Joshi, 2010*(30) |
| 19 | China | NS | NS | 5 | 25.90 | 22.80 ~ 30.00 | *Cao, 2010*(31) |
|  |  | Soil fertilization | 15 kg·hm^-2^ ZnSO_4_·7H_2_O | 5 | 24.10 | 23.40 ~ 24.60 | *Cao, 2010*(31) |
|  |  | Foliar fertilization | 1.5 kg·hm^-2^ ZnSO_4_·7H_2_O | 5 | 42.20 | 38.50 ~ 46.00 | *Cao, 2010*(31) |
|  |  | Soil and foliar fertilization | 15 kg·hm^-2^ and 1.5 kg·hm^-2^ ZnSO_4_·7H_2_O | 5 | 46.90 | 43.50 ~ 49.40 | *Cao, 2010*(31) |
| 20 | Turkey | Soil and foliar fertilization | 0,50,80,240 kg·hm^-2^ and 0.5% ZnSO_4_·7H_2_O | 1 | 36.63 | 10.40 ~ 70.00 | *Cakmak, et al,. 2010*(33) |
| 21 | China | Soil fertilization | NS | 112 | 33.83 | 23.94 ~ 52.89 | *Zhang, et al., 2011*(29) |
| 22 | Turkey | Soil fertilization | 3.1 kg·hm^-2^ Zn | NS | 21.50 | 8.00 ~ 40.00 | *Cakmak, 2011*(27) |
| 23 | China | Soil fertilization | 0, 7.5, 15, 30, 45 kg·hm^-2^ Zn | 10 | 25.74 | 22.80 ~ 28.80 | *Li, 2011*(28) |
| 24 | India | NS | NS | 37 | 33.70 | 28.50 ~ 44.10 | *Velu, 2012*(26) |
| 25 | Pakistan | NS | NS | 37 | 31.80 | 29.30 ~ 34.20 | *Velu, 2012*(26) |
| 26 | Mexico | NS | NS | 37 | 30.40 | 26.80 ~ 34.00 | *Velu, 2012*(26) |
| 27 | China | NS | NS | NS | 23.11 | NS | *Wang, 2012*(5) |
|  |  | Soil fertilization | 50 kg·hm^-2^ ZnSO_4_·7H_2_O | NS | 29.11 | NS | *Wang, 2012*(5) |
|  |  | Foliar fertilization | 4 kg·hm^-2^ ZnSO_4_·7H_2_O | NS | 35.59 | NS | *Wang, 2012*(5) |
|  |  | Soil and foliar fertilization | 50 kg·hm^-2^ and 4 kg·hm^-2^ ZnSO_4_·7H_2_O | NS | 43.61 | NS | *Wang, 2012*(5) |
| 28 | India | NS | NS | 10 | 19.76 | 10.53 ~ 25.10 | *Bharti, et al., 2013*(25) |
|  |  | Soil fertilization | 20 kg·hm^-2^ ZnSO_4_· | 10 | 22.07 | 15.40 ~ 31.27 | *Bharti, et al., 2013*(25) |
|  |  | Soil and foliar fertilization | 20 kg·hm^-2^ and 0.5% ZnSO_4_· | 10 | 33.59 | 25.13 ~ 42.57 | *Bharti, et al., 2013*(25) |
| 29 | China | NS | NS | 40 | 29.67 | 21.98 ~ 42.00 | *Zhang, et al., 2014*(24) |
| 30 | Mexico | Soil fertilization | NS | 22 | 21.70 | 15.80 ~ 24.30 | *Guzmán, et al., 2014*(51) |
| 31 | China | Soil fertilization | NS | 3 | 22.37 | 20.40 ~ 24.40 | *Li, et al., 2015*(22) |
|  |  | Foliar fertilization | 3g·L^-1^ ZnSO_4_·7H_2_O | 3 | 38.73 | 32.90 ~ 43.00 | *Li, et al., 2015*(22) |
|  |  | Soil and foliar fertilization | 3 g·L^-1^ ZnSO_4_·7H_2_O | 6 | 41.52 | 30.00 ~ 47.60 | *Li, et al., 2015*(22) |
| 32 | United States | NS | NS | 4 | 47.50 | NS | *Guttieri, et al., 2015*(21) |
| 33 | United States | Soil fertilization | NS | NS | 28.23 | 18.90 ~ 56.10 | *Guttieri, et al., 2015*(21) |
| 34 | China | NS | NS | NS | 28.85 | NS | *Liu, 2016*(19) |
|  |  | Soil fertilization | 20 kg·hm^-2^ ZnSO_4_·7H_2_O | NS | 33.33 | NS | *Liu, 2016*(19) |
|  |  | Seed soaking | 3 g·L^-1^ ZnSO_4_·7H_2_O | NS | 29.49 | NS | *Liu, 2016*(19) |
|  |  | Soil and seed soaking | 20 kg·hm^-2^ and 3 g·L^-1^ ZnSO_4_·7H_2_O | NS | 34.31 | NS | *Liu, 2016*(19) |
| 35 | Iran | NS | NS | NS | 18.80 | NS | *Sadeghzadeh, 2016*(18) |
|  |  | Foliar fertilization | 2 g·L^-1^ ZnSO_4_·7H_2_O | 7 | 37.07 | 25.10 ~ 47.50 | *Sadeghzadeh, 2016*(18) |
| 36 | China | Foliar fertilization | 1.2 kg·hm^-2^ ZnSO_4_· | 2 | 41.80 | 38.20 ~ 45.60 | *Zhang, 2016*(20) |
| 37 | China | Foliar fertilization | 5 g·L^-1^ ZnSO_4_·7H_2_O | 40 | 55.00 | 39.30 ~ 88.20 | *Zhang, 2017*(15) |
| 38 | India | NS | NS | 6 | 36.50 | 33.70 ~ 39.50 | *Saha et al., 2017*(16) |
| 39 | Mexico | Soil fertilization | NS | 46 | 37.20 | 31.80 ~ 48.80 | *Magallanes-López et al., 2017*(17) |
| 40 | China | Soil fertilization | 50 kg·hm^-2^ ZnSO_4_·7H_2_O or 100 kg·hm^-2^ ZnSO_4_·7H_2_O | NS | 45.50 | 34.00 ~ 57.00 | *Liu, 2017*(10) |
| 41 | China | NS | NS | 6 | 35.20 | 33.40 ~ 37.10 | *Huang, et al., 2018*(13) |
| 42 | Pakistan | Soil fertilization | 10 kg·hm^-2^ ZnSO_4_ | 28 | 33.80 | 21.20 ~ 54.40 | *Rehman, et al., 2018*(14) |
| 43 | China | Soil fertilization | NS | 2 | 30.70 | 22.40 ~ 39.60 | *Abdul Rashid, 2019*(12) |
| 44 | India | NS | NS | 1 | 30.20 | 25.20 ~ 38.80 | *Abdul Rashid, 2019*(12) |
| 45 | Zambia | NS | NS | 1 | 29.00 | 24.00 ~ 34.20 | *Abdul Rashid, 2019*(12) |
| 46 | Pakistan | NS | NS | 3 | 28.40 | 25.20 ~ 31.00 | *Abdul Rashid, 2019*(12) |
| 47 | Mexico | NS | NS | 19 | 34.30 | 24.80 ~ 43.70 | *Kaur, et al., 2020*(11) |
|  | Mean |  |  |  | 31.84 | 8.00 ~ 88.20 |  |

Note: NS indicates that relevant information is not mentioned in the literature or no zinc fertilizer is applied.

**Table S2** Zinc content in different components of wheat

| **Serial number** | **Country** | **Peeling rate (%)** | **Flour extraction rate (%)** | **Number of samples** | **Zinc content in wheat flour (mg·kg^-1^)** | | **Zinc content in wheat Shorts (mg·kg^-1^)** | | **Zinc content in wheat bran (mg·kg^-1^)** | | **References** |
| --- | --- | --- | --- | --- | --- | --- | --- | --- | --- | --- | --- |
|  |  |  |  |  | **Mean** | **range** | **Mean** | **range** | **Mean** | **range** |  |
| 1 | United States | NS | 60.00 | 27 | 6.80 | 5.30 ~ 8.70 | NS | NS | 73.50 | 51.50 ~ 80.70 | *Peterson, 1983*(46) |
| 2 | France | NS | NS | 7 | 6.10 | 3.20 ~ 10.40 | NS | NS | NS | NS | *Wang, 1998*(45) |
|  | China | NS | NS | 5 | 10.70 | 4.60 ~ 14.70 | NS | NS | NS | NS | *Wang, 1998*(45) |
| 3 | Pakistan | 0 | 100.00 | 6 | 24.40 | 21.00 ~ 27.50 | NS | NS | NS | NS | *Anjum, et al., 2002*(68) |
|  |  | NS | NS |  | 20.10 | 9.00 ~ 33.50 | NS | NS | 64.80 | 43.50 ~ 80.50 | *Anjum, et al., 2002*(68) |
| 4 | Belgium | NS | 69.00 | 14 | 11.60 | 2.70 ~ 36.50 | 69.67 | 53.30 ~ 87.70 | 78.10 | 52.80 ~ 134.10 | *Ruibal-Mendieta, et al., 2005*(42) |
| 5 | China | NS | 50.00 ~ 85.00 | 3 | 6.23 | 5.20 ~ 7.8 | NS | NS | NS | NS | *Ma, 2005*(67) |
| 6 | China | 0 | 73 | 4 | 6.60 | 4.60 ~ 9.00 | 38.80 | 26.60 ~ 51.70 | NS | NS | *Liu, et al., 2008*(71) |
|  |  | 5.00 ~ 15.00 | 78.00 ~ 86.00 | 4 | 5.97 | 4.50 ~ 7.70 | 41.20 | 27.60 ~ 52.00 | 37.65 | 23.80 ~ 54.90 | *Liu, et al., 2008*(71) |
| 7 | China | 10.20 ~ 17.30 | 61.20 ~ 70.30 | 43 | 8.40 | 5.40 ~ 15.50 | 49.10 | NS | 86.30 | NS | *Tang, et al., 2008*(38) |
| 8 | Italy | NS | 66.00 | 3 | 11.30 | 10.30 ~ 13.30 | NS | NS | NS | NS | *Cubadda, et al., 2009*(37) |
| 9 | China | NS | 78.00 | 1 | 13.80 | 11.20 ~ 16.90 | 70.30 | 54.30 ~ 83.50 | 82.90 | 60.00 ~ 102.80 | *Shi, et al., 2010*(66) |
| 10 | China | NS | 76.00 | 2 | 17.40 | 10.90 ~ 18.40 | 94.30 | 67.60 ~ 133.60 | 111.10 | 77.90 ~ 159.80 | *Zhang, et al., 2010*(32) |
| 11 | China | NS | NS | 39 | 8.45 | 5.69 ~ 15.06 | NS | NS | 60.93 | 47.51 ~ 85.85 | *Zhang, et al., 2011*(29) |
| 12 | Pakistan | NS | NS | 12 | 13.00 | 7.10 ~ 30.00 | NS | NS | NS | NS | *Akhte, et al,. 2012*(65) |
| 13 | China | NS | 60.00 ~ 65.00 | 5 | 9.80 | 9.50 ~ 11.30 | NS | NS | NS | NS | *Zhang, et al., 2012*(64) |
| 14 | United States | NS | 76.00 ~ 78.00 | 4 | 17.50 | NS | NS | NS | 131.00 | NS | *Guttieri, et al., 2015*(21) |
| 15 | Bolivia | NS | NS | NS | 15.20 | 11.50 ~ 17.80 | NS | NS | NS | NS | *Lazarte, et al., 2015*(55) |
| 16 | China | NS | 65.00 ~ 75.00 | 1 | 13.00 | 8.20 ~ 17.40 | NS | NS | 70.30 | 36.40 ~ 98.50 | *Li, et al., 2015*(22) |
| 17 | China | 0 | NS | 3 | 10.10 | 7.50 ~ 12.60 | NS | NS | NS | NS | *Zou, et al., 2015*(63) |
|  |  | 2.00 ~ 12.00 | NS | 3 | 8.72 | 5.50 ~ 16.40 | NS | NS | NS | NS | *Zou, et al., 2015*(63) |
| 18 | United States | NS | NS | NS | 7.80 | 7.80 ~ 8.70 | 91.00 | 91.00 ~ 98.00 | 91.00 | 91.00 ~ 106.00 | *Brier, et al., 2015*(62) |
|  |  | 3.00 ~ 12.00 | NS | NS | 8.38 | NS | 96.00 | NS | 102.00 | NS | *Brier, et al., 2015*(62) |
| 19 | China | NS | 65.00 ~ 75.00 | 1 | 19.40 | 11.50 ~ 24.50 | NS | NS | 134.30 | 86.10 ~ 154.00 | *Li, et al., 2016*(61) |
| 20 | Ethiopia | 0 | 100.00 | 2 | 30.30 | 24.70 ~ 35.90 | NS | NS | NS | NS | *Heshe, et al., 2016*(60) |
|  |  | NS | 68.00 | 2 | 9.90 | 5.80 ~ 13.90 | NS | NS | NS | NS | *Heshe, et al., 2016*(60) |
| 21 | China | NS | 75.40 | 1 | 8.30 | 6.10 ~ 13.50 | 87.57 | 51.28 ~ 139.84 | 81.83 | 51.58 ~ 137.12 | *Zhang, et al,. , 2017*(59) |
| 22 | Italy | 0 | 100.00 | 4 | 33.95 | NS | NS | NS | NS | NS | *Ciccolini, et al., 2017*(58) |
|  |  | NS | NS | 4 | 7.50 | NS | NS | NS | NS | NS | *Ciccolini, et al., 2017*(58) |
| 23 | China | NS | 66.00 ~ 78.00 | 3 | 11.06 | 7.99 ~ 13.47 | NS | NS | NS | NS | *Gao, 2018*(57) |
| 24 | China | NS | NS | 3 | 8.31 | 7.59 ~ 9.09 | 66.91 | 64.17 ~ 69.98 | 90.99 | 87.49 ~ 97.03 | *Gong, et al., 2019*(56) |
|  | Mean |  |  |  | 12.58 | 2.70 ~ 36.50 | 70.49 | 26.60 ~ 139.84 | 86.45 | 23.80 ~ 154.00 |  |

Note: NS indicates that relevant information is not mentioned in the literature
