# Supplementary material for: Zinc in Wheat Grain, Processing, and Food
Source: Front Nutr. 2020 Aug 18;7:124. doi: 10.3389/fnut.2020.00124 (PMC7471629; doi:10.3389/fnut.2020.00124)
Supplement: Supplementary file 3 [file Image_1.pdf]

Zinc in wheat grain, processing, and food

Min Wang, Fanmei Kong, Rui Liu, Qingqi Fan, Xiaocun Zhang

Supplementary Figures

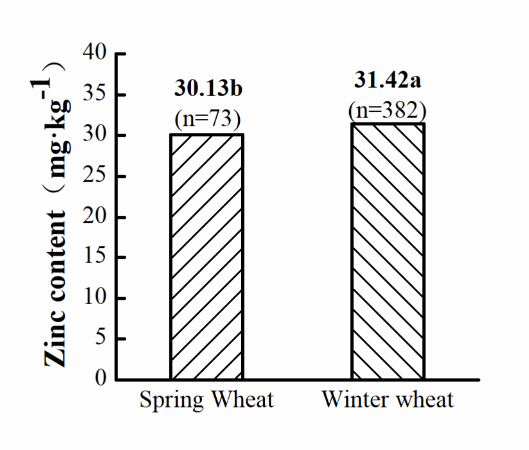

Figure S1 Zinc content in spring and winter wheat

Note: The letters a and b indicate a significant difference at the level of $P < 0.05$; n is the total number of samples.

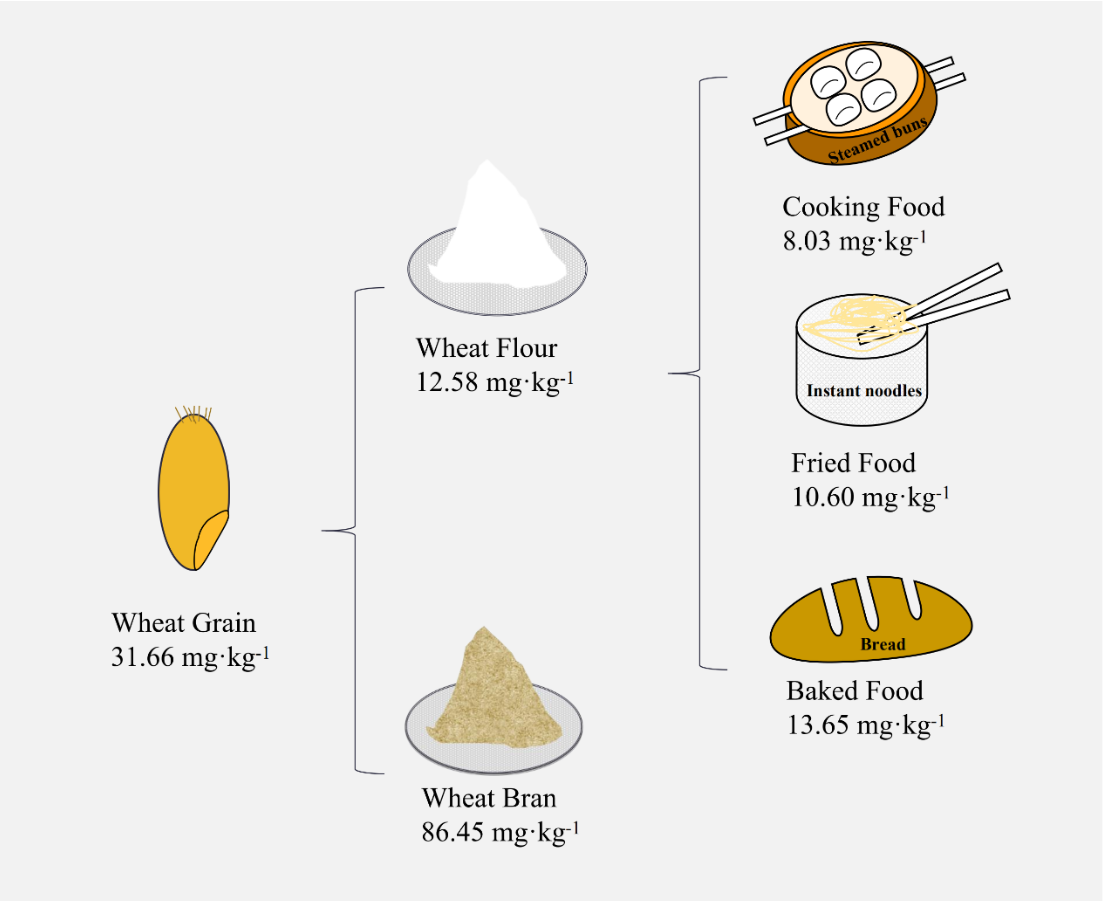

Figure S2 Distribution of Zn in wheat grain and its processing foods
